# Higher TLR7 Gene Expression Predicts Poor Clinical Outcome in Advanced NSCLC Patients Treated with Immunotherapy

**DOI:** 10.3390/genes12070992

**Published:** 2021-06-29

**Authors:** Sara Baglivo, Fortunato Bianconi, Giulio Metro, Alessio Gili, Francesca Romana Tofanetti, Guido Bellezza, Biagio Ricciuti, Martina Mandarano, Valeria Teti, Annamaria Siggillino, Maria Sole Reda, Rita Chiari, Lorenza Pistola, Angelo Sidoni, Vincenzo Minotti, Fausto Roila, Vienna Ludovini

**Affiliations:** 1 Medical Oncology Division, Santa Maria della Misericordia Hospital, Piazzale Menghini 8/9, 06132 Perugia, PG, Italy; giulio.metro@ospedale.perugia.it (G.M.); francesca.tofanetti@ospedale.perugia.it (F.R.T.); annamaria.siggillino@ospedale.perugia.it (A.S.); mariasole.reda@ospedale.perugia.it (M.S.R.); lorenza.pistola@ospedale.perugia.it (L.P.); vincenzo.minotti@ospedale.perugia.it (V.M.); fausto.roila@ospedale.perugia.it (F.R.); vienna.ludovini@ospedale.perugia.it (V.L.); 2 Umbria Digitale, Regional Government of Umbria, Via G.B. Pontani 39, 06128 Perugia, PG, Italy; fortunato.bianconi@gmail.com; 3 Public Health Section, Department of Experimental Medicine, University of Perugia, Piazza Lucio Severi 1, 06132 Perugia, PG, Italy; alessio.gili@gmail.com; 4 Section of Anatomic Pathology and Histology, Department of Medicine and Surgery, University of Perugia, Piazza Lucio Severi 1, 06132 Perugia, PG, Italy; guido.bellezza@unipg.it (G.B.); martina.mandarano@unipg.it (M.M.); valeria.teti83@hotmail.com (V.T.); angelo.sidoni@unipg.it (A.S.); 5 Lowe Center for Thoracic Oncology, Dana-Farber Cancer Institute, Boston, MA 02215, USA; biagio.ricciuti@gmail.com; 6 Division of Medical Oncology, Ospedali Riuniti Padova Sud, Via Albere 30, 35043 Monselice, PD, Italy; rita.chiari@aulss6.veneto.it

**Keywords:** non-small-cell lung cancer (NSCLC), predictive biomarkers, immune gene expression, immunotherapy, immune checkpoint inhibitor (ICI), PD-L1, Toll-like receptors (TLRs)

## Abstract

Immune checkpoint inhibitors (ICIs) have revolutionized the treatment of lung cancer. However, their clinical benefit is limited to a minority of patients. To unravel immune-related factors that are predictive of sensitivity or resistance to immunotherapy, we performed a gene expression analysis by RNA-Seq using the Oncomine Immuno Response Assay (OIRRA) on a total of 33 advanced NSCLC patients treated with ICI evaluating the expression levels of 365 immune-related genes. We found four genes (CD1C, HLA-DPA1, MMP2, and TLR7) downregulated (*p* < 0.05) and two genes (IFNB1 and MKI67) upregulated (*p* < 0.05) in ICI-Responders compared to ICI-Non-Responders. The Bayesian enrichment computational analysis showed a more complex interaction network that involved 10 other genes (IFNA1, TLR4, CD40, TLR2, IL12A, IL12B, TLR9, CD1E, IFNG, and HLA-DPB1) correlated with different functional groups. Five main pathways were identified (FDR < 0.0001). High TLR7 expression levels were significantly associated with a lack of response to immunotherapy (*p* < 0.0001) and worse outcome in terms of both PFS (*p* < 0.001) and OS (*p* = 0.03). The multivariate analysis confirmed TLR7 RNA expression as an independent predictor for both poor PFS (HR = 2.97, 95% CI, 1.16–7.6, *p* = 0.023) and OS (HR = 2.2, 95% CI, 1–5.08, *p* = 0.049). In conclusion, a high TLR7 gene expression level was identified as an independent predictor for poor clinical benefits from ICI. These data could have important implications for the development of novel single/combinatorial strategies TLR-mediated for an efficient selection of “individualized” treatments for NSCLC in the era of immunotherapy.

## 1. Introduction

In recent years, immune checkpoint inhibitors (ICIs) have revolutionized the therapeutic landscape of many cancer types, including non-small-cell lung cancer (NSCLC) [1], significantly improving patient outcomes. Several ICIs (anti-CTLA-4, anti-PD-1, and anti-PD-L1) are able to restore the antitumor immune response and have been approved by the U.S. Food and Drug Administration (FDA) and the European Medicines Agency (EMA) for therapy in advanced NSCLC and other solid tumors. The anti-PD-1 agent pembrolizumab is approved for use as first- and second-line therapy in patients with advanced NSCLC expressing PD-L1 in an immunohistochemical analysis [2,3]. Pembrolizumab has shown survival benefits in the frontline setting for patients with metastatic NSCLC as a single-agent monotherapy and with platinum-based agents in combination therapy, depending on PD-L1 expressions ≥50% and <50%, respectively. Nivolumab (anti-PD-1) and atezolizumab (anti-PD-L1) are both indicated for use as second-line therapies regardless of PD-L1 expression [4,5]. Such immunotherapeutic drugs can interfere with immune cells in the tumor microenvironment, restoring the activity of antitumor T cells and facilitating control of the tumor [6].

Despite unprecedented progress, much remains to be learned regarding how best to leverage these new agents in oncology practices. Unfortunately, only a fraction of patients benefits significantly from immunotherapy, with most patients not achieving an objective response. It is noteworthy that the expression of PD-L1 is not always a predictor of efficacy of PD-1 or PD-L1 inhibitors, and conversely, evidence has shown that some patients can respond even to low or absent PD-L1 expression [7]. In addition to the urgent need for an international standard method for a PD-L1 expression analysis, there is a compelling need for a better understanding of the factors that could potentially predict the response and resistance to immunotherapy. Newer advanced technologies can be used to explore and evaluate the tumor microenvironment and the complexities of tumor and immune system interactions, going beyond the assessment of single analytes such as PD-L1. Recent studies have suggested that Tumor Mutational Burden (TMB), Microsatellite Instability and Mismatch Repair Deficiency, assessment of the Tumor Immune Microenvironment (TME) by T-cell tumor infiltration level evaluations, and gene expression profiles (GEPs) can correlate with the clinical response to immunotherapy [8,9,10]. Recent data have also suggested that immune-related gene signatures may predict the clinical response to immunotherapy. Ayers et al. [10] identified immune-related signatures that correlated with the clinical benefits from ICIs using a learn-and-confirm paradigm based on data from different clinical studies of pembrolizumab. Both genomic and transcriptomic features of tumors can contribute to the response to an immune checkpoint blockade. Recent genomic mutational studies have highlighted the driver genetic alterations that underlie the tumor immune response. Anti-PD-1/PD-L1 therapy does not achieve a significant survival improvement in patients with *EGFR* mutations [11,12,13] or with co-occurring *KRAS/STK11* alterations [14]. The search for biomarkers able to predict the response to an immune checkpoint blockade is becoming increasingly important for patient selection. As the use of ICIs also in first-line therapy is rapidly changing the treatment scenario for advanced NSCLC, robust predictive biomarkers might prove critical for therapeutic decisions, especially in the case of reliable negative predictive factors, which may potentially allow selection of those patients who do not benefit from the use of an ICI in addition to or in place of platinum-based chemotherapy, irrespective of PD-L1 expression.

The study of the tumor microenvironment (TME) by a transcriptomic analysis could shed light on the primary cancer immune resistance that represents one of the critical challenges in immuno-oncology. To this aim, in the present study, we investigated the immune-related transcriptomic landscape of advanced NSCLC tumors to understand the predictors of the immune response to help identify the patients most likely to benefit from immunotherapy.

## 2. Materials and Methods

### 2.1. Patient Selection

The present study included a retrospective cohort of patients with advanced NSCLC who received a second or a subsequent line therapy of an anti-PD-1 inhibitor (nivolumab) at the Medical Oncology Division of Santa Maria della Misericordia Hospital in Perugia, Italy during the period from July 2015 to June 2019. A total of 43 eligible patients were initially identified. The medical records of the patients were retrospectively reviewed. Only patients with available clinical data and tumor tissues were considered to be eligible. All tumor samples were collected before ICI treatment. Ten specimens were excluded because of their poor quality and unsuitability for the gene expression analysis. The study was approved by the local ethical committee “CEAS UMBRIA” (Comitato Etico Aziende Sanitarie Umbria) with code number 2870/16 and was conducted in accordance with ethical principles of the latest version of the Declaration of Helsinki. Written informed consent for the gene expression analyses was obtained from each patient recruited in the study.

### 2.2. RNA Sequencing by Oncomine Immune Response Research Assay

RNA-sequencing was performed at the Molecular Biology Laboratory of Medical Oncology Division of S, Maria della Misericordia Hospital in Perugia, Italy, using the Oncomine Immune Response Research Assay (OIRRA) panel (Thermo Fisher Scientific, Carlsbad, CA, USA) to sensitively measure the expression levels of 395 genes associated with 36 functional groups, including checkpoint pathways, lymphocyte regulation, cytokine interactions, lymphocyte, and tumor marker. For RNA extraction, 2–5 sections of 10 µm-thick formalin-fixed paraffin-embedded (FFPE) tissues were prepared. One slide at the beginning of each serial section was stained with hematoxylin-eosin (H&E) and histopathologically examined by a board-certified pathologist to determine the tumor cell contents. After macrodissection of the tumor area, RNA was isolated using the RNeasy FFPE Kit (Qiagen GmbH, Hilden, Germany) on a QiaCube robotic workstation according to the manufacturer’s instructions. RNA concentrations were determined with the Qubit RNA HS Assay Kit and a Qubit 3.0 Fluorometer (Thermo Fisher Scientific, Carlsbad, CA, USA). RNA quality assessment was performed by a real-time qPCR functional RNA quantitation (FRQ) assay. NGS libraries were prepared automatically on the Ion Chef™ system (Thermo Fisher Scientific) using the OIRRA-Chef Ready panel and 10 ng of RNA previously reverse-transcribed into cDNA through the SuperScript VILO™ cDNA synthesis kit (Thermo Fisher Scientific). A diluted 50-pM single pool of cDNA libraries, quantified by qPCR with the Ion Library TaqMan^®^ Quantitation Kit, was used for the sequencing of the 395 immune-related genes. Template preparation and enrichment were automatically performed using the Ion Chef™ system and the Ion PGM Hi-Q View Chef Kit. The sequencing step was performed on the Ion PGM System using the Ion 318™ Chip v2 (all from Thermo Fisher Scientific). Alignment of the sequences to the reference immuneresponse_V3.1 and counting of the sequencing reads and normalized gene level counting data were performed using the ImmuneResponseRNA plug-in in Torrent Suite software. A gene-level differential expression analysis was performed with Transcriptome Analysis Console (TAC) 4.0 software.

### 2.3. PD-L1 Testing

Immunohistochemistry (IHC) for PD-L1 was performed at the Section of Anatomic Pathology and Histology of Perugia University, Italy, using the PD-L1 22C3 pharmDx kit (Dako North America Inc., Carpinteria, CA, USA) on the Dako Autostainer Link 48 according to the manufacturer’s instructions. Unstained tissue sections 4-m-thick were prepared from the most representative area of each case. At least 100 viable tumor cells were required for a valid interpretation of PD-L1 staining. Slides were counterstained with Mayer’s hematoxylin. Results were evaluated with known positive and negative tissue controls. The percentage of PD-L1 expression in the invasive tumor cells was calculated as the number of viable invasive carcinoma cells showing membranous staining of any intensity divided by the total number of viable invasive carcinoma cells.

### 2.4. Bayesian Network Analysis

A gene network analysis based on the Bayesian algorithm was performed by GeneMANIA database querying with the genes selected through a RNA expression analysis. We predicted the genes associated with RNA expression analysis and their interaction networks using a linear regression-based algorithm that calculates a single composite functional association network based on multiple data sources and a label propagation algorithm that was used to predict the gene function given the composite functional association network. Predicted genes were scored based on their relevance to the original query genes [15]. The *Homo sapiens* database of GeneMANIA was used (updated on 17 March 2017). We performed query using GO biological process parameters.

### 2.5. Statistical Analysis

The gene-level expression results obtained from the sequencing run were normalized by Read Per Million (RPM) and downloaded from Torrent Suite software to be uploaded on TAC 4.0 software. The gene expression levels by the univariate ANOVA analysis were considered upregulated or downregulated with fold changes >2 or <−2, respectively, and a *p*-value < 0.05. Gene expression levels were assessed as continuous and categorical variables. We used the median values of each gene as the cut-off to discriminate low and high gene expression levels. Patients were divided into two groups based on their clinical responses to ICI, according to the Response Evaluation Criteria in Solid Tumor (RECIST) v1.1. Patients showing their best response, complete response, partial response, or stable disease >6 months were considered as Responder (ICI-R), while those patients showing disease progression or the stable disease lasting ≤6 months were considered as Non-Responders (ICI-Non-R).

Descriptive statistics were calculated, including frequencies, percentages, frequency tables for categorical variables, and mean ± standard deviation (SD) or median for quantitative variables. The categorical variables were evaluated by Χ² or Fisher’s exact test when appropriate.

The objectives were to describe the clinical outcome in terms of the overall (whole body) response rate (ORR, meaning complete + partial responses), disease control rate (DCR, meaning complete + partial responses + stable disease), and DCB (meaning complete + partial responses + stable disease >6 months and corresponding to a percentage of ICI-Responders) in all patients and according to their gene expression. Progression-free survival (PFS) was defined as the time in months from the date of the first dose of ICI treatment to first disease progression at any site. Overall survival (OS) was defined as the time in months from the date of first dose of the anti-PD-1 inhibitor until death from any cause. The Kaplan–Meier method was used to analyze the PFS and OS and estimate the medians with two-sided 95% confidence intervals (CI).

Survival curves were compared using the log-rank test. Cox’s regression models (univariate and multivariate) were applied to estimate the Hazard Ratio and 95% CI and to identify the prognostic factors independently associated with the survival times. All factors with statistically significant results from the univariate approach were included in the final multivariate model.

Significance was set at ≤0.05 in all tests. Statistical analyses were performed with STATA v. 16.1 (StataCorp LP, College Station, TX, USA).

## 3. Results

### 3.1. Patients

A total of 33 Stage IV NSCLC patients were included in the study. All patients were treated at the Medical Oncology Division of Santa Maria della Misericordia Hospital in Perugia with a single agent immune checkpoint inhibitor after at least one anticancer therapy in the metastatic setting. The clinicopathological characteristics of the patients are reported in Table 1. The majority of patients received one or two previous lines of treatments (*n* = 19, 57.6%; *n* = 11, 33.3%, respectively). The median age at the beginning of ICI treatment was 67 years (range, 46–84); 67% (*n* = 22) were male, and 82% (*n* = 27) had a former or current smoking history. The performance status was 0 in 10 (30%) patients and 1 or 2 in 14 (42%) and nine (27%) patients, respectively. The histological subtypes included 23 adenocarcinomas (70%) and 23 squamous cell carcinomas (30%). PD-L1 expression according to the IHC evaluation was <1% in 21 (63.6%) patients and ≥1% in 12 (36.4%) patients, of which 10 patients encompassed 1–49% (30.3%) and only two patients (6.1%) >50%. At the time of the last follow-up (15 March 2021), four patients (12%) were still alive.

### 3.2. Tumor Response Evaluation

Table 2 shows the clinical response to treatment with ICI according to the RECISTv1.1 criteria. Of 33 patients, 10 patients (30%) and four patients (12.1%) experienced a partial response (PR) and stable disease (SD), respectively, while 19 patients (57.6%) showed progressive disease (PD) as their best response. At the time of the last follow-up, one patient who obtained PR was still on treatment with ICI (24 months).

The objective response rate was 30.3% (10 of 33 patients), and the disease control rate (DCR) was 42.4% (14 of 33 patients). Of the 33 patients, 13 (39.4%) achieved a durable clinical benefit (ICI-R), and the remaining 20 (60.6%) patients showed no durable benefit (ICI-Non-R).

We next compared ICI-R and ICI-Non-R for clinicopathological characteristics. Patients with a durable clinical benefit were significantly more likely to be former/current smokers compared to ICI-Non-R (*p*= 0.029). The predictive biomarker PD-L1 expression by IHC was also compared between the two groups, and no significant difference was observed (*p* = 0.09).

### 3.3. RNA-Seq and Immune-Related Gene Expression Analyses

**Figure 1:**
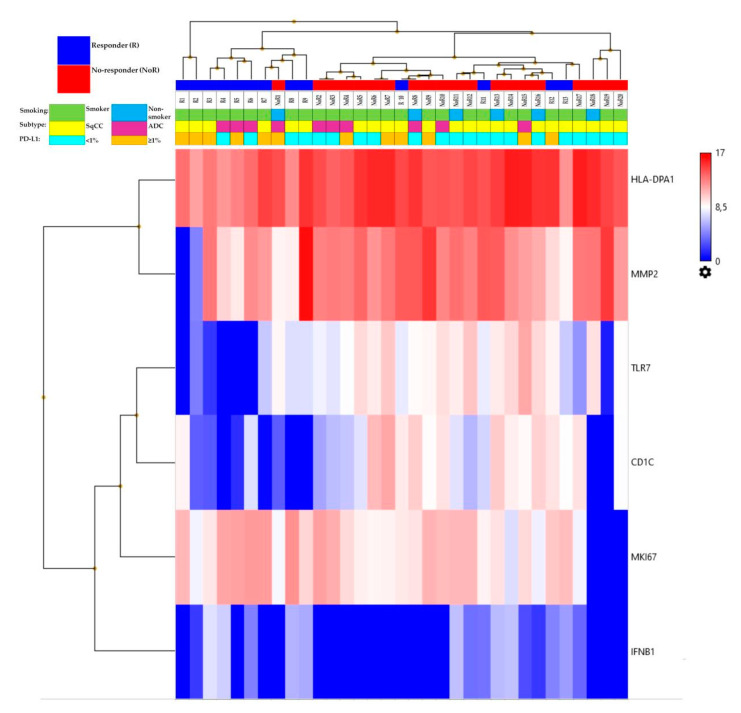
Heatmap of the genes differentially expressed between ICI-Responders and ICI-Non-Responders.

We investigated the immune-related gene expression landscape using the OIRRA, obtaining successful transcriptomic data from 33 cases of advanced NSCLC. To identify the transcriptomic factors associated with the response to immunotherapy, patients were divided in two groups based on their clinical response to ICI: Responder (*n* = 13) and Non-Responder (*n* = 20) groups (Table 3). The gene expression level analysis by the univariate ANOVA test revealed six genes differentially expressed between the two groups: four genes (CD1C, HLA-DPA1, MMP2, and TLR7) downregulated (*p* < 0.05) and two genes (IFNB1 and MKI67) upregulated (*p* < 0.05) in ICI-Responders compared to ICI-Non-Responders, as reported in Table 4. Figure 1 shows the heatmap of the genes differentially expressed in the two groups. The six genes belonged to different functional groups: antigen presentation (HLA-DPA1 and CD1C), innate immune response (TLR7), type II interferon signaling (IFNB1), tumor marker (MMP2), and proliferation marker (MKI67).

We evaluated the gene expression levels of the six genes of the signature identified by the ANOVA analysis and their association with the clinicopathological characteristics of the patients. A high expression level of HLA-DPA1 was significantly associated with adenocarcinoma histology (*p* = 0.03), whereas the expression level of IFNB1 was significantly lower in male patients (*p* = 0.05). A low expression level of TLR7 was significantly associated with smokers (*p* = 0.005). No further statistically significant correlations were observed.

**Figure 2:**
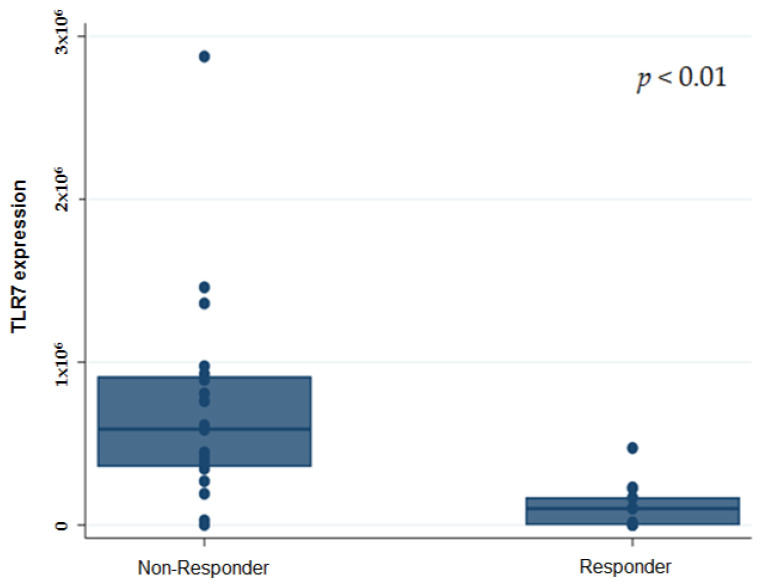
Box plot for TLR7 gene expression according to ICI-Responders and ICI-Non-Responders.

Furthermore, we investigated the association of the gene expression analysis with ORR, DCR, and DCB. Both ORR and DCR were lower in patients with a high RNA ex-pression of TLR7 (*p* = 0.004 and *p* < 0.0001, respectively). Of the 16 patients with a high gene expression level of TLR7, 93.7% (15/16) were Non-Responders, with only 6.25% of the patients obtaining a durable clinical benefit (DCB, 6.25% vs. 93.75%, *p* < 0.0001). Seventy-one percent of the patients with a low gene expression level of TLR7 were re-sponder patients (DCB 70.6% vs. 29.4; *p* < 0.0001) (Figure 2).

We further analyzed the association between TLR7 gene expression and PDL-1 IHC expression and response to ICI. Of the 15 ICI-Non-Responders with high TLR7 expression, 27% (4/15) were PD-L1 positive (*p* = 0.7). Of the 12 ICI-Responders with a low TLR7 expression, 50% (6/12) and 50% (6/12) showed PD-L1 expressions <1% and ≥1%, respectively (*p* = 0.3).

### 3.4. Survival Analyses and Correlation with Immune-Related Gene Expression

At a median follow-up of 11.7 months (range 1–66), the median PFS was 3.3 months (95% CI 1.9–6.6), while the median OS was 11.7 months (95% CI 6.4–21.8). At the univariate analysis, PS = 1 to 2 before ICI treatment, no smoking history, and PD-L1 IHC expression <1% were significantly associated with shorter PFS (*p* = 0.014, *p* = 0.013, and *p* = 0.022, respectively) (Table 5 and Figure S1), whereas PS = 1 to 2 and squamous histology (*p* = 0.007 and *p* = 0.006, respectively) were significantly associated with a worse OS (Table 6).

To unravel the immune-related factors that are predictive of a sensitivity or resistance to immunotherapy, a survival analysis in association with the gene expression level was performed.

**Figure 3:**
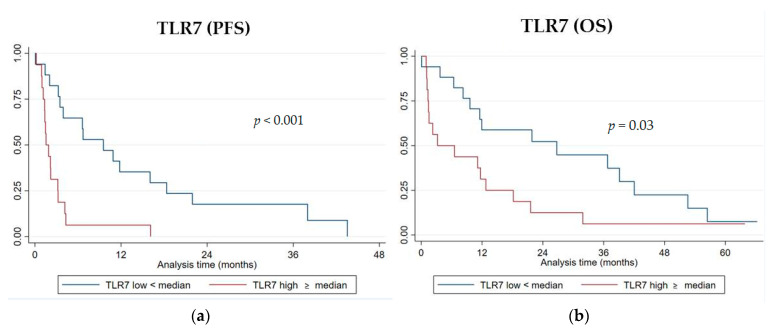
Kaplan–Meyer survival curves for progression-free survival (PFS) (**a**) and overall survival (OS) (**b**) according to TLR7 gene expression.

Among the six genes of the signature investigated, the RNA level expression of TLR7 was significantly associated with both PFS and OS. Patients with a high expression level of TLR7 (higher than the median value) displayed a worse PFS compared to those patients with a low one (lower than the median value) (median PFS 1.5 vs. 9.5 months, log-rank *p* < 0.001 (Figure 3a); hazard ratio HR = 3.94, 95% CI, 1.77–8.75, *p* = 0.001) (Table 5).

A high TLR7 RNA expression was significantly associated with a shorter OS (median OS 3.2 vs. 26.7 months, log-rank *p* = 0.03 (Figure 3b); HR = 2.2, 95% CI 1.05–4.68, *p* = 0.036) (Table 6). No statistically significant differences in PFS or OS were observed with high or low gene expression levels for CD1C, HLA-DPA1, MMP2, IFNB1, and MKI67.

A multivariate Cox’s regression model for PFS and OS was performed using the variables that were found significant at the univariate analysis. In the multivariate analysis, a PS = 1 to 2 and PD-L1 <1% remained significantly associated with a worse PFS (HR= 5.4, 95% CI 1.8–16.1, *p* = 0.002 and HR= 4.1, 95% CI 1.58–10.9, *p* = 0.004, respectively), whereas squamous histology remained significantly associated with a shorter OS (HR = 3.6, 95% CI, 1.3–9.8, *p* = 0.013). The TLR7 RNA expression independently predicted both the PFS and OS, with high gene expression levels significantly associated with a worse PFS (HR = 2.97, 95% CI, 1.16–7.6, *p* = 0.023) and worse OS (HR = 2.2, 95% CI, 1–5.08, *p* = 0.049).

### 3.5. Bayesian Network Analysis

**Figure 4:**
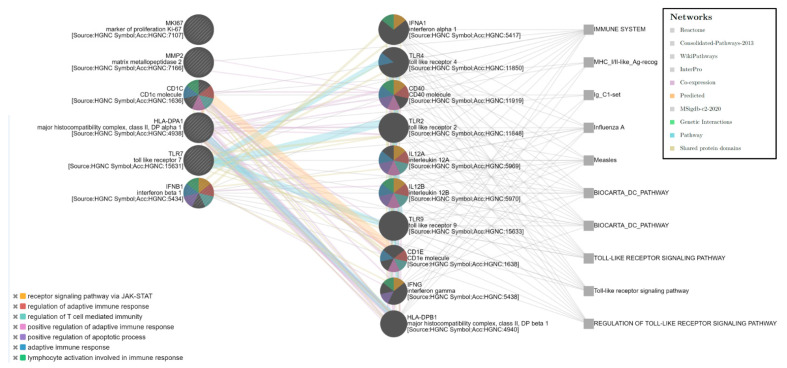
Gene correlation network by the Bayesian computational analysis.

The Bayesian enrichment computational analysis of the six-gene expression signatures (HLA-DPA1, TLR7, MMP2, CD1C, IFNB1, and MKI67) showed a more complex network that involved 10 other genes (IFNA1, TLR4, CD40, TLR2, IL12A, IL12B, TLR9, CD1E, IFNG, and HLA-DPB1) correlated with different functional groups also related to the immune response. Five main pathways were identified (False Discovery Rate; FDR < 0.0001), such as regulation of the adaptative immune response and T-cell-mediated immunity, lymphocyte activation, regulation of the apoptotic process, and receptor signaling pathway via JAK-STAT, which involves five of the six genes (all except MKI67) among the gene expression signature identified (Figure 4). The network analysis highlighted the correlation between TLR7 and other TLR family members (TLR2, TLR4, and TLR9) and, also, with other immune modulatory molecules.

## 4. Discussion

In the present study, we evaluated the mRNA expression data to identify significant transcriptomic features associated with the immune response to ICI in patients affected by metastatic NSCLC. A gene expression analysis by RNA-Seq revealed a six-gene expression signature differentially expressed between Responder and Non-Responder patients to immunotherapy. The six genes of the signature belonged to different functional immune-related groups: antigen presentation (HLA-DPA1 and CD1C), innate immune response (TLR7), type II interferon signaling (IFNB1), tumor marker (MMP2), and proliferation marker (MKI67). The Bayesian enrichment computational analysis revealed a more complex network that involved 10 other genes (IFNA1, TLR4, CD40, TLR2, IL12A, IL12B, TLR9, CD1E, IFNG, and HLA-DPB1) correlated with different functional groups. Five pathways mainly involved in immune regulatory functions were identified.

Among the six genes, the expression levels of TLR7 were able to discriminate Responder and Non-Responder patients to ICI. We found high expression levels of TLR7 significantly associated with a lack of response to immunotherapy. Patients with high gene expression levels of TLR7 experienced a worse outcome in terms of both PFS and OS.

The Bayesian network analysis showed the correlation of TLR7 with several immune modulatory molecules and other TLR family members, such as TLR2, TLR4, and TLR 9. The cancer systems biology analysis approach strengthened our data identifying an immune molecular network and confirmed the correlation of the gene expression signature with relevant immune regulatory functions.

Toll-like receptors (TLRs) are a class of pattern recognition receptors (PRRs) that recognize a variety of molecules from invading pathogens, so-called “pathogen-associated molecular patterns” (PAMPs), and activate innate immunity and inflammatory responses [16]. TLRs play a critical role in both innate and adaptive immunity but are also expressed on a wide variety of tumors regulating tumor growth and functions [17].

The role of TLRs in cancer is complex and controversial. An increased expression or activation of these receptors may result in pro- or antitumorigenic effects, depending on the molecular context. Increasing evidence suggests that the activation of TLRs in immune cells or tumor cells promotes an antitumor immune response or induces tumor immune evasion [18,19]. TLR signaling affects the differentiation and function of different T-cell subsets and can directly reverse the suppressive function of tumor-derived CD4+, CD8+, and γδ Treg cells [20]. By contrast, the activation of tumor cell TLRs can promote tumor cell proliferation and resistance to apoptosis and enhance tumor cell invasion and metastasis by regulating metalloproteinases and integrins. Furthermore, the activation of TLR signaling in tumor cells induces the synthesis of the proinflammatory factors and immunosuppressive molecules, which promote the resistance of tumor cells to cytotoxic lymphocyte attacks, leading to immune evasion [21]. TLRs can regulate hypoxia-derived metabolites such as cAMP and IDO, which are potent immune suppressors, as TLR7 stimulation significantly increases the IDO expression. More specifically, TLR7 is an endosomal receptor for single-stranded RNA expressed on dendritic cell macrophage B lymphocytes and NK cells. The stimulation of these cells with TLR7 ligands induces their maturation and activation and the secretion of proinflammatory cytokines, mainly through activation of the NF-KB pathways. Contrary to the therapeutic benefits of TLR7 agonists on the immune cells, two studies have shown that TLR7 can be highly expressed on primary tumor cells from NSCLC patients, and its stimulation on tumor cells promotes tumor progression and resistance to chemotherapy treatment in NSCLC patients who highly express TLR7. The protumorigenic effect could be mediated either by the direct stimulation of TLR7-expressing tumor cells or by the increased recruitment of immunosuppressive cells in the TME, such as myeloid-derived suppressor cells (MDSCs) and a reduction of CD8 T cells [22,23].

In the current study, we showed that, in advanced NSCLCs treated with ICI, the gene expression level of TLR7 strongly associates with a poor clinical efficacy. To the best of our knowledge, our study is the first to demonstrate a relationship between TLR7 and the immune response to ICI, with a strong correlation between TLR7 RNA expression and a poor clinical outcome. As previous studies have reported, our results confirmed the potential protumorigenic effects of TLR7 in opposition to the antitumor role that this receptor can interplay. TLR7 in this context could promote an immune suppressive microenvironment through the stimulation of proinflammatory factors and regulation of immunosuppressive molecules, which protect cancer cell from the T-cell-mediated antitumor immune response. Unfortunately, in our study, a TILS assessment, which might have reinforced our findings, was not feasible.

Tumor immune evasion may be facilitated by inhibitory cytokines, inflammatory factors, and immunosuppressive molecules. Inflammation mediates tumor-induced tolerance. Immune tolerance in cancer mediates tumor escape from the immune system. The activation of TLRs in tumor cells induce the synthesis of proinflammatory factors and immunosuppressive molecules. We hypothesized that TLR7 could promote an immune suppressive microenvironment that enhances the resistance of tumor cells and promotes immune evasion.

The signaling pathways that trigger a tumor cell escape from immune surveillance are not completely understood. In our study, the PD-L1 level assessed by IHC was identified as an independent predictor for PFS. However, there was no correlation between the RNA expression of TLR7 and PD-L1 IHC expression. Furthermore, 50% of ICI-Responders with low TLR7 RNA expression and 27% percent of ICI-Non-Responders with high TLR7 expression were PD-L1-positive (>1%).

Our findings suggest that both in the presence or absence of the expression of PD-L1, despite the inhibition of PD-1/PD-L1 signaling mediated by ICI, the high levels of TLR7 may facilitate the evasion of immune surveillance, maintaining a suppressive microenvironment and promoting resistance to ICI treatment.

As TLRs can act as a double-edged sword with both protumorigenic and antitumor effects, understanding the functional regulations in tumor cells and tumor-infiltrating immune cells mediated by TLRs will be important for the success of TLR-based cancer immunotherapies and will force us to rethink the role and therapeutic potential of TLR signaling, considering that most current TLR-involved treatments have been disappointing in clinical trials.

This study had several limitations. Due to its retrospective design and the small size of the population investigated, a potential selection bias could not be excluded. Our study was based on mRNA gene expression, which could be affected by inter- and-intra-tumoral heterogeneity and did not consider the protein level expression. Furthermore, our research was limited to a specific immune-related set of genes investigated by the multiplex panel compared to a wide transcriptomic analysis.

Further studies with a prospective design on a larger population are necessary to validate our results and to better understand the relationship between TLRs and the TME for the development of better anticancer therapies. Multiple features are involved in the response to ICI; thus, novel and multiple biomarkers are needed to potentially capture the complexity of the TME. A TMB assessment could also be investigated in relation to specific molecular pathways and gene expressions as a multidimensional approach for patient stratification with immunotherapy to better select patients who would most likely benefit from treatment. Correlations between genetics and transcriptomic data with outcomes of clinical interest also increase the biological knowledge of cancer.

Understanding the complex interactions between the host immune system and cancer molecular biology could lead to a comprehensive tool that can be useful to drive treatments and clinical patient managements. Immune checkpoint inhibitors could be only the tip of the iceberg in the discovery and development of targeted therapies exploiting the immune system to fight cancer. As novel biomarkers are being identified, many other immune targets such as TLR7 could be used to develop new drugs beyond ICI that modulate the immune system, with drug combinations potentially eliciting a better immune response than single drugs.

## 5. Conclusions

In conclusion, in this study, we performed a gene expression analysis to identify the significant genes associated with the sensitivity or resistance to ICI. The gene expression level of TLR7 was identified as an independent predictor for poor clinical benefits from ICI. Further studies that confirm our research and evaluate combined biomarkers are needed. A multidimensional approach enabling a comprehensive molecular characterization of cancer TME could be the key for an efficient selection of “individualized” treatment strategies and patient management in order to realize precision medicine for NSCLC in the era of immunotherapy.

## Figures and Tables

**Table 1:** Clinicopathological characteristics of the patients.

Characteristics	Patients	
	*n* = 33	%
**Median Age,** years (range)	67 (46–84)	
**Gender**		
Female	11	33.3
Male	22	66.7
**Smoking History**		
Never smokers	6	18
Former/Current smokers	27	82
**Histology**		
Squamous cell carcinomas	10	30.3
Adenocarcinomas	23	69.7
Performance Status *		
0	10	30
1	14	42
2	9	27
**N. lines of therapy before ICI**		
1	19	57.6
2	11	33.3
≥3	3	9.1
**PD-L1 status**		
<1%	21	63.6
1–49%	10	30.3
≥50%	2	6.1
Genetic alterations **		
EGFR, p.E746_A750del	1	3
EGFR, p.L858R	1	3
EGFR, p.S768_D770dup+p.P772T	1	3
**Exitus**		
Live	29	88
Dead	4	12

* Before ICI treatment; ** adenocarcinoma histology.

**Table 2:** Response to treatment with the immune checkpoint inhibitor.

Best Response *	Total *n* = 33 (%)
Partial response (PR)	10 (30.3)
Stable disease (SD)	4 (12.1)
Progressive disease (PD)	19 (57.6)
Objective Response (Rate) (CR + PR)	10 (30.3)
Disease Control (Rate) (CR + PR + SD)	14 (42.4)

* Assessed by RECISTv1.1.

**Table 3:** Responder and Non-Responder patients according to their clinical responses to ICI.

Clinical Response to ICI *	Total *n* = 33 (%)
ICI-Responder (ICI-R) **	13 (39.4)
ICI-Non-Responder (ICI-Non-R) ***	20 (60.6)

* Assessed by RECISTv1.1; ** ICI-Responder: CR/PR/SD > 6 months; *** ICI-Non-Responder: PD/SD ≤6 months.

**Table 4:** Differentially expressed genes between ICI-Responders and ICI-Non-Responders.

Genes	Fold Change (linear) (ICI-R vs. ICI-Non-R)	ANOVA *p*-Value	Gene Function
HLA-DPA1	−2.73	0.000239	Antigen presentation
TLR7	−12.39	0.000262	Innate immune response
MMP2	−4.13	0.017494	Tumor marker
CD1C	−20.25	0.027213	Antigen presentation
IFNB1	13.39	0.037871	Type II interferon signaling
MKI67	2.04	0.038822	Proliferation

Abbreviations: HLA-DPA1, major histocompatibility complex class II DP alpha 1; TLR7, Toll-like receptor 7; MMP2, matrix metallopeptidase 2; CD1C, CD1c molecule; IFNB1, interferon-beta 1; and MKI67, marker of proliferation Ki-67.

**Table 5:** Univariate and multivariate Cox analyses for progression-free survival (PFS).

	PFS					
	Univariate Analysis			Multivariate Analysis		
**Variables**	HR	95% IC	p*	HR	95% IC	p*
Age **	0.98	0.94–1.05	0.4	-	-	-
Sex, male vs. female	0.7	0.36–1.64	0.5	-	-	-
Smoking, never vs. ever ***	3.4	1.29–9.00	**0.013**	3.05	0.88–10.5	0.077
Histology, squamous vs. adeno	1.6	0.73–3.68	0.22	-	-	-
PS, 1–2 vs. 0	2.98	1.24–7.17	**0.014**	5.4	1.8–16.1	**0.002**
PD-L1, <1% vs. ≥1%	2.5	1.14–5.56	**0.02**	4.1	1.58–10.9	**0.004**
TLR7, high vs. low	3.9	1.77–8.75	**0.001**	2.97	1.15–7.61	**0.023**
CD1C, high vs. low	1.7	0.83–3.53	0.14	-	-	-
HLA-DPA1, high vs. low	1.03	0.5–2.13	0.93	-	-	-
IFNB1, low vs. high	1.5	0.73–3.1	0.26	-	-	-
MMP2, high vs. low	1.77	0.87–3.6	0.11	-	-	-
MKI67, low vs. high	1.65	0.8–3.4	0.17	-	-	-

Abbreviations: PFS, Progression-free Survival; HR, Hazard Ratio; CI, Confidence Interval; PD-L1, programmed cell death 1 ligand 1; PS, performance status; HLA-DPA1, major histocompatibility complex class II DP alpha 1; TLR7, Toll-like receptor 7; MMP2, matrix metallopeptidase 2; CD1C, CD1c molecule; IFNB1, interferon-beta 1; and MKI67, marker of proliferation Ki-67. p*-value ≤ 0.05 in bold; ** assessed as a continuous variable; and *** current/former smokers.

**Table 6:** Univariate and multivariate Cox analyses for overall survival (OS).

	OS					
	Univariate Analysis			Multivariate Analysis		
**Variables**	HR	95% IC	p*	HR	95% IC	p*
Age **	1.01	0.97–1.05	0.49	-	-	-
Sex, male vs. female	1.58	0.69–3.61	0.27	-	-	-
Smoking, never vs. ever ***	1.34	0.5–3.58	0.54	-	-	-
Histology, squamous vs. adeno	3.36	1.42–7.97	0.006	3.6	1.31–9.84	0.01
Performance status, 1–2 vs. 0	3.34	1.39–8	**0.007**	2.28	0.83–6.29	0.10
PD-L1, <1% vs. ≥1%	1.34	0.61–2.91	0.45	-	-	-
TLR7, high vs. low	2.20	1.05–4.68	**0.036**	2.2	1.00–5.08	**0.049**
CD1C, low vs. high	1.17	0.5–2.46	0.66	-	-	-
HLA-DPA1, low vs. high	1.36	0.65–2.87	0.4	-	-	-
IFNB1, low vs. high	1.38	0.66–2.88	0.38	-	-	-
MMP2, high vs. low	1.12	0.54–2.35	0.74	-	-	-
MKI67, high vs. low	1.01	0.48–2.13	0.96	-	-	-

Abbreviations: OS, Overall Survival; HR, Hazard Ratio; CI, Confidence Interval; PD-L1, programmed cell death 1 ligand 1; PS, performance status; HLA-DPA1, major histocompatibility complex class II DP alpha 1; TLR7, Toll-like receptor 7; MMP2, matrix metallopeptidase 2; CD1C, CD1c molecule; IFNB1, interferon-beta 1; and MKI67, marker of proliferation Ki-67. p*-value ≤ 0.05 in bold; ** assessed as continuous variable, and *** current/former smokers.

## Data Availability

Not applicable.

## Supplementary Materials

The following are available online at https://www.mdpi.com/article/10.3390/genes12070992/s1: Figure S1. Kaplan–Meyer survival curves for progression-free survival (PFS) according to PD-L1 IHC expression.
