# Supplementary figures and images for: Higher TLR7 Gene Expression Predicts Poor Clinical Outcome in Advanced NSCLC Patients Treated with Immunotherapy
Source: Genes (Basel). 2021 Jun 29;12(7):992. doi: 10.3390/genes12070992 (PMC8303258; doi:10.3390/genes12070992)

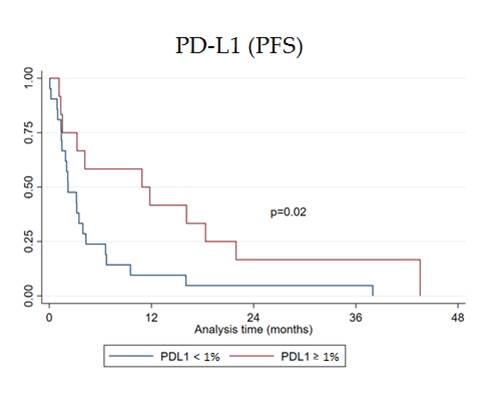

Supplement: Supplementary file 1 [file genes-12-00992-s001.zip › Fig S1/Fig. S1.jpg]
